# Unilateral Biportal Endoscopic en Bloc Resection of the Lumbosacral Ligament Following Bony Release: A Technique Aiming to Reduce Dorsal Root Ganglion Manipulation

**DOI:** 10.3390/jcm15103765

**Published:** 2026-05-14

**Authors:** Cheng-Ying Lee, Cheol-Wung Park, Man-Kyu Park, Wei-Yu Lee, Chien-Min Chen

**Affiliations:** 1 Department of Neurosurgery, Neurological Institute, Taichung Veterans General Hospital, Taichung 407, Taiwan; cklove5212@gmail.com; 2 Department of Neurosurgery, Daejeon Woori Hospital, Daejeon 35262, Republic of Korea; endospine@naver.com; 3 Department of Neurosurgery, Hu Hospital, Busan 47392, Republic of Korea; jihak3@hanmail.net; 4 Department of Neurosurgery, Dajia Lee General Hospital, Taichung 437, Taiwan; leeweiyu@ms9.hinet.not; 5 Division of Neurosurgery, Department of Surgery, Changhua Christian Hospital, Changhua 500, Taiwan; 6 Department of Biomedical Sciences, National Chung Cheng University, Chiayi 621, Taiwan

**Keywords:** unilateral biportal endoscopy, far-out syndrome, lumbosacral ligament

## Abstract

**Background**: Far-Out Syndrome at the lumbosacral junction is caused by extraforaminal compression of the L5 nerve root, frequently involving the lumbosacral ligament (LSL). Conventional piecemeal resection of the LSL may increase the risk of postoperative dysesthesia due to repeated manipulation near the L5 dorsal root ganglion (DRG). This study introduces a novel unilateral biportal endoscopic (UBE) technique for en bloc resection of the LSL. **Methods**: The technique is based on an osteoclastic release strategy in which the bony attachments of the LSL, including the inferior aspect of the L5 transverse process and the sacral ala, are drilled and released before addressing the ligament itself. This maneuver elevates the LSL away from the underlying L5 DRG and achieves en bloc removal under direct endoscopic visualization. **Results**: En bloc resection may improve visualization within the narrow extraforaminal corridor and may reduce direct mechanical manipulation of the L5 DRG, which could potentially translate into reduced postoperative dysesthesia. The presented technique enabled effective decompression without repeated instrument insertion beneath the ligament. **Conclusions**: UBE-assisted en bloc resection of the LSL is a feasible and potentially neuroprotective technique for treating lumbosacral extraforaminal lesions.

## 1. Introduction

The evolutionary trajectory of spinal surgery has been continuously shaped by the anatomical complexities of the lumbosacral junction. Extraforaminal stenosis at the L5-S1 level, frequently referred to in the neurosurgical literature as Far-Out Syndrome (FOS), was initially described by Wiltse et al. in 1984 [1]. Wiltse accurately identified that the L5 spinal nerve root can become severely entrapped lateral to the intervertebral foramen, compressed both dynamically and statically between the transverse process (TP) of the L5 vertebra and the sacral ala. Unlike typical central canal stenosis or lateral recess stenosis—which are predominantly driven by ligamentum flavum hypertrophy or central disc herniation within the spinal canal—FOS is distinctly multifactorial and exists outside the traditional boundaries of the spinal canal.

To fully understand the mechanical constraints of FOS, a comprehensive understanding of the extraforaminal architecture—specifically the “lumbosacral tunnel”—is required. The lumbosacral tunnel extends from the extraforaminal zone of the L5-S1 intervertebral foramen to the sacral ala. The anatomical boundaries of this highly constrained space include the anterior–superior surface of the sacral ala forming the floor, the lateral aspect of the L5 vertebral body and intervertebral disc defining the medial boundary, the inferior surface of the L5 transverse process serving as the proximal roof, and the lumbosacral ligament (LSL) forming the lateral border and roof [2].

Pathophysiological studies have consistently demonstrated that compression in FOS is multifactorial, involving pseudoarticulation, osteophyte formation, disc bulging, and, importantly, thickening of the lumbosacral and extraforaminal ligaments [3].

The LSL is a robust, dense fibrous structure that plays an important role in stabilizing the lumbosacral junction. Originating from the inferior cortical border of the L5 TP, the ligament extends obliquely and inferolaterally to reach the sacral ala. Superiorly, the LSL is partially continuous with the inferior margin of the iliolumbar ligament, and inferomedially, it blends with the anterior sacroiliac ligament [4]. Biomechanically, the LSL acts in concert with the iliolumbar ligament to restrict excessive flexion, extension, and lateral bending at the L5-S1 junction. The L5 spinal nerve root traverses this tunnel, passing over the sacral ala and dorsal to the LSL. In addition, the sympathetic ramus communicans to the L5 root typically penetrates the superior border of the LSL to join the nerve within this tunnel, accompanied by branches of the iliolumbar vessels. In pathological conditions, the LSL may undergo hypertrophy and fibrosis, transforming from a stabilizing structure into a potential compressive element that reduces the available extraforaminal space. Therefore, adequate neural decompression in FOS may require careful resection of this thickened ligamentous structure [5]. Adequate decompression may depend on the removal of the thickened ligamentous structure.

A critical neurosurgical consideration at this level is the L5 dorsal root ganglion (DRG). The L5 DRG is among the largest in the lumbar spine and is frequently located in the extraforaminal zone, making it potentially susceptible to injury [6]. Anatomical studies utilizing magnetic resonance imaging (MRI) and cadaveric dissection have shown that approximately 95% of L5 DRGs are located within the intraforaminal or immediate extraforaminal zone, placing them within the operative corridor required to access and treat FOS [2]. Mechanical manipulation, excessive static retraction, or repetitive microtrauma to the DRG during surgical decompression may contribute to postoperative dysesthesia (POD) [7]. POD is a neurogenic condition characterized by persistent burning pain, hyperalgesia, allodynia, and sensory disturbances in the corresponding dermatome, which may adversely affect the perceived benefit of decompression [8]. The contemporary literature suggests that POD following transforaminal or extraforaminal endoscopic decompression may occur in up to one-fifth of patients, with extraforaminal stenosis and severe foraminal narrowing identified as potential risk factors for increased dysesthesia rates [7].

Conventional endoscopic or microscopic piecemeal resection typically involves repeated insertion of a Kerrison punch and curettes beneath the LSL to fragment and remove the ligament. This approach may lead to repeated mechanical contact with the underlying DRG and could increase the risk of iatrogenic neural irritation, as suggested by previous studies on similar decompression techniques [9].

To address the limitations of piecemeal resection in this confined, neurovascularly rich zone, we propose a UBE technique for en bloc resection of the LSL. This approach is conceptually adapted from en bloc resection techniques described for the ligamentum flavum, which emphasize the release of bony attachments prior to ligament removal and may help reduce neural manipulation while improving procedural efficiency [10].

To the best of our knowledge, the use of en bloc resection of the LSL for the treatment of lumbosacral extraforaminal lesions has not been previously reported. The aims of this technical modification are to minimize manipulation of the L5 DRG, optimize the surgical working space, and facilitate adequate decompression of the L5 nerve root in the extraforaminal zone.

## 2. Materials and Methods

The surgical procedure is demonstrated in Supplementary Video S1.

### 2.1. Anesthesia, Positioning, and Portal Targeting

**Figure 1:**
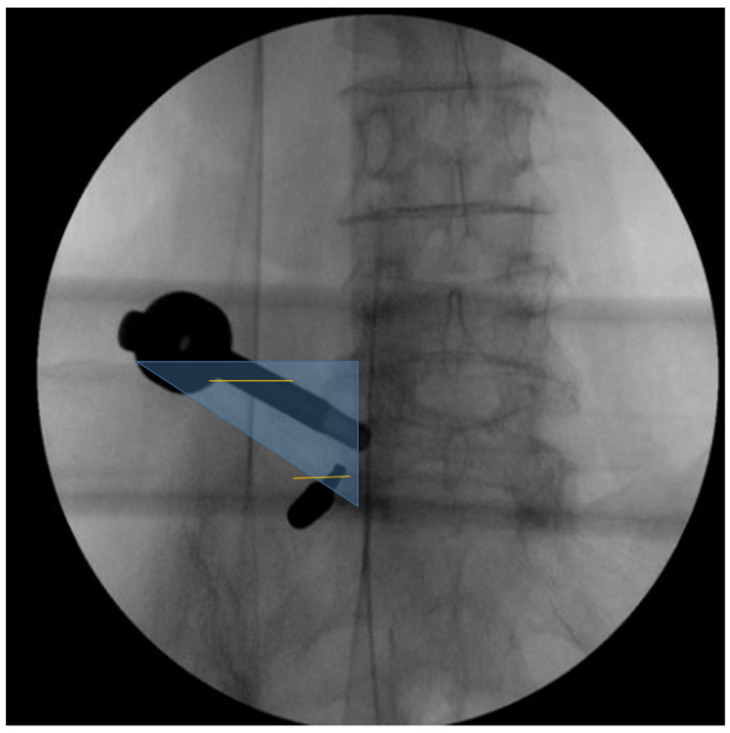
Intraoperative fluoroscopic anteroposterior (AP) view demonstrating surgical portal planning. The translucent blue triangle indicates the triangular corridor formed by the L5-S1 lateral pedicle line and the iliac crest. The two horizontal lines indicate the planned positions for the surgical incisions.

The procedure is performed under general anesthesia. Patients are positioned prone on a radiolucent table with chest and pelvic bolsters to allow abdominal suspension and reduce intra-abdominal pressure, thereby minimizing epidural venous congestion. Adequate padding is applied to all pressure points to prevent position-related complications. Intraoperative neuromonitoring was not routinely used. The target is the superior sacral notch, defined radiographically as the junction between the lateral aspect of the S1 superior articular process (SAP) and the sacral ala. UBE requires two portals using a paraspinal approach. The cranial portal (endoscopic viewing portal) is typically placed approximately 2 cm lateral to the L5 lateral pedicle line or at the distal end of the L5 TP. If this entry point falls outside the triangular working corridor, it is adjusted medially to the nearest feasible position within the corridor to optimize access. The caudal portal serves as the working portal for instrument manipulation and is located medial to the iliac crest at the level of the superior sacral notch, with an approximately 1 cm skin incision (Figure 1).

### 2.2. Hydrodynamic Regulation and Soft Tissue Clearance

Upon insertion of the endoscope (Stryker, Kalamazoo, MI, USA) and working sheaths, continuous normal saline irrigation is established. Fluid pressure management is an important safety parameter in UBE procedures. The hydrostatic pressure generated by the irrigation pump (Stryker CrossFlow Arthroscopy Pump, Kalamazoo, MI, USA) is maintained between 25 and 30 mmHg. This pressure range provides adequate hydrostatic tamponade for hemostasis while maintaining a clear visual field without exceeding safe epidural pressure thresholds. Exceeding these thresholds may increase the risk of retroperitoneal fluid accumulation if the anterior fascial boundaries are violated.

**Figure 2:**
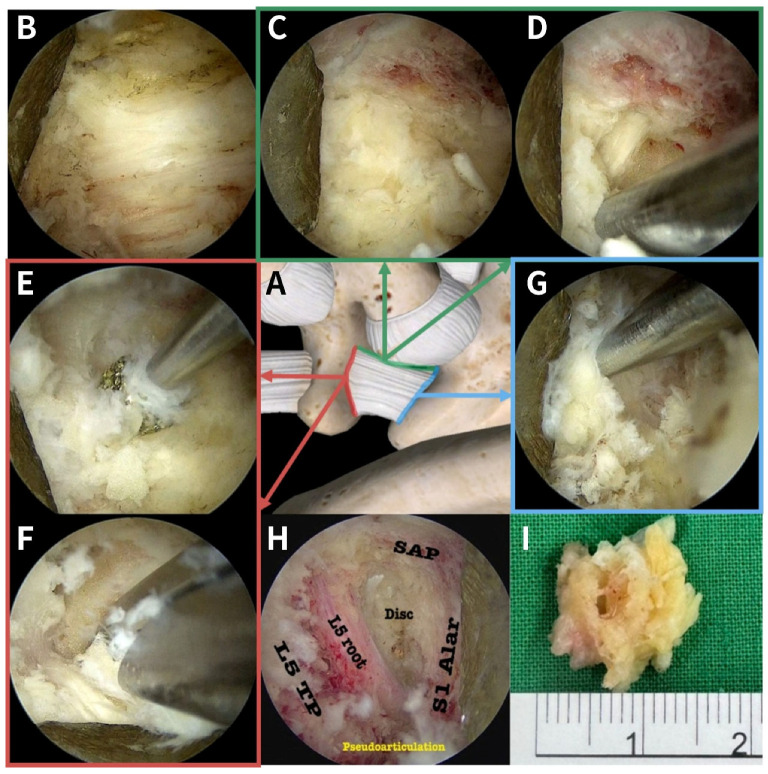
Unilateral biportal endoscopic en bloc resection of the LSL. (**A**) Three-dimensional schematic of the L5-S1 extraforaminal junction showing the LSL and its bony attachments: superiorly to the L5 TP inferior border (red line); medially to the S1 SAP (green line); and inferiorly to the S1 Ala (blue line). (**B**) Endoscopic view of the thick, fibrotic LSL complex prior to bony work. Osteoclastic release (**C**–**G**): The rigid LSL attachments are systematically released using a high-speed diamond burr (**C**,**E**) and Kerrison punch (**D**,**F**,**G**) to detach the ligament from the SAP (**C**,**D**), the L5 TP (**E**,**F**), and the Sacral Ala (**G**). This maneuver separates the LSL from its bony anchors, allowing it to be suspended away from the L5 DRG. (**H**) Final endoscopic visualization of the decompression corridor after en bloc removal of LSL. The L5 nerve root is completely mobilized, lying free over the Disc and S1 Alar floor. (**I**) Gross pathology specimen demonstrating the LSL removed as a single en bloc unit.

Initial soft tissue dissection and clearance over the key osseous landmarks are performed using a radiofrequency (RF) plasma wand (Apollo, Arthrex, Naples, FL, USA), including 90-degree and ball-type probes. The RF device is used for coagulation and ablation of the extraforaminal epidural venous plexus and accompanying branches of the iliolumbar artery prior to mechanical dissection. The primary objective of this phase is to expose the key bony landmarks, including the lateral border of the S1 SAP, the inferior cortical margin of the L5 TP, and the superior aspect of the sacral ala (Figure 2A,B).

### 2.3. The Osteoclastic Release Phase

The defining characteristic of this en bloc technique is the surgical isolation of the LSL through systematic osteoclastic release of its bony attachments, separating the ligament from its rigid skeletal anchors prior to resection. This technically demanding process is performed in three sequential phases using a high-speed coarse diamond burr (NSK, Tochigi, Japan) under continuous endoscopic visualization with irrigation to minimize thermal injury:

Medial Release: The lateral aspect of the S1 SAP is drilled to its base. This maneuver reshapes the articular process and releases the LSL from its medial constraints, disconnecting it from the joint capsule (Figure 2C,D).

Cranial Release: The high-speed burr is advanced superiorly to thin and resect the inferior cortical margin of the L5 TP (Figure 2E), facilitating detachment of the cranial origin of the LSL (Figure 2F).

Caudal and Lateral Release: The burr is then directed inferiorly to thin the superior edge of the sacral ala. In cases where an osteophytic pseudoarticulation (LSTV Castellvi type II/IV) is present, the osteophytes are resected to expand the working space and establish the floor of the extraforaminal decompression zone (Figure 2G).

Throughout the osteoclastic release, decompression proceeds in a medial-to-lateral direction along the ligament’s attachment. Cancellous bone bleeding encountered during drilling is controlled using an RF probe or endoscopic bone wax.

### 2.4. Establishment of Extraforaminal Space and LSL Suspension

Following detachment of its medial, cranial, and caudal osseous attachments, the biomechanical properties of the LSL are altered, resulting in reduced ligament tension. At this stage, the ligament remains attached primarily to residual deep extraforaminal soft tissue and the anterior fascial layer. In this partially detached state, the LSL is positioned above the underlying L5 nerve root and the DRG.

This step facilitates expansion of the extraforaminal space. Using a bimanual technique characteristic of biportal endoscopy, the surgeon introduces an endoscopic retractor through the viewing portal to gently elevate the LSL dorsally. This maneuver creates a clear, tension-reduced working plane between the ligament and the underlying L5 DRG.

### 2.5. En Bloc Resection and Complete Ventral Decompression

With the LSL elevated and the DRG visualized, a cutting instrument, such as a Kerrison rongeur or an arthroscopic basket punch, is introduced through the working portal. Because the ligament is detached and lifted, the footplate of the Kerrison rongeur does not need to be advanced into a confined space ventral to the nerve root. The LSL can then be resected as a single piece (en bloc) or in large segments without direct mechanical compression of the DRG.

Following resection of the LSL, the extraforaminal and far-lateral course of the L5 nerve root becomes more clearly visualized. The surgeon then assesses the ventral aspect of the tunnel. Any residual ventral pathology contributing to FOS, such as extraforaminal disc herniation from the L5-S1 intervertebral space or projecting vertebral body osteophytes, can be accessed and removed using standard UBE discectomy instruments and pituitary rongeurs. The expanded working space facilitates ventral decompression. The endpoint of the procedure is confirmed when the L5 nerve root and DRG appear adequately decompressed, with visible mobility and pulsation within the irrigation field from the neuroforaminal exit to the distal extraforaminal zone (Figure 2H).

The LSL was removed en bloc (Figure 2I).

### 2.6. Postoperative Care

Following completion of the procedure and placement of an optional subfascial closed-suction drain, patients are transferred to the recovery unit. Early mobilization is encouraged, and patients are typically permitted to ambulate with a supportive brace on the day of surgery or early on postoperative day 1, reflecting the tissue-sparing nature of the UBE approach.

## 3. Results

### 3.1. Case Illustration

A 65-year-old male presented with a chronic, debilitating history of radicular pain in the left L5 distribution. The patient’s symptoms were refractory to conservative management, including a structured 6-month regimen of pharmacological therapy and physical rehabilitation. Physical examination confirmed radiculopathy consistent with left L5 nerve root compression.

### 3.2. Imaging Findings

**Figure 3:**
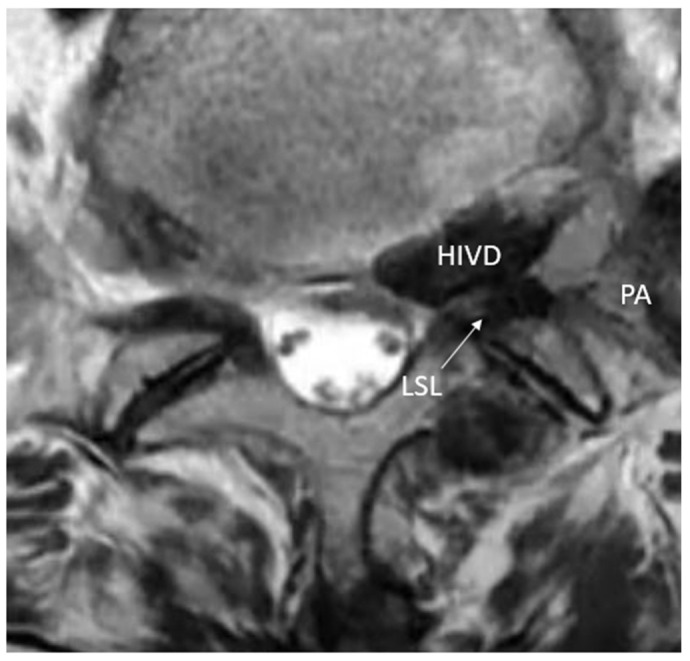
Preoperative axial T2-weighted MRI at the L5-S1 level. The image demonstrates significant entrapment of the left L5 nerve root within the extraforaminal space. It clearly shows compression due to the herniated disc, hypertrophied lumbosacral ligament, and the formation of a pseudoarticulation. HIVD: herniated intervertebral disc; LSL: lumbosacral ligament; PA: pseudoarticulation.

Preoperative MRI revealed significant extraforaminal entrapment of the left L5 nerve root at the L5-S1 level. The compression was attributed to a herniated intervertebral disc (HIVD), hypertrophied LSL, and the pseudoarticulation of L5 TP and S1 Alar (Figure 3). Based on these findings, FOS was diagnosed.

### 3.3. Surgical Intervention

Given the failure of six months of conservative treatment, the patient underwent UBE decompression. The surgical goals were to achieve comprehensive decompression of the L5 nerve root while preserving spinal stability. Intraoperatively, the hypertrophied LSL, the offending portion of the HIVD, and the pseudoarticulation were successfully resected.

### 3.4. Results and Follow-Up

**Figure 4:**
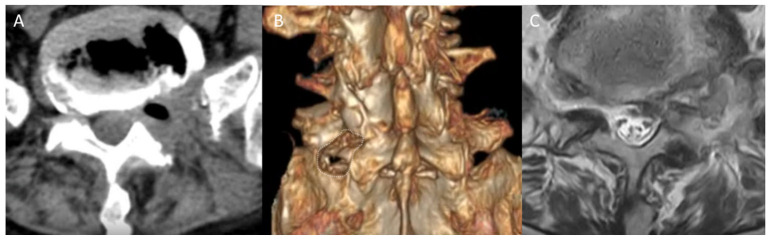
Postoperative imaging evaluation. (**A**) Axial CT scan showing the extent of the bony decompression. (**B**) Three-dimensional CT reconstruction; the white dotted line delineates the area where intraoperative bone resection (bony work) was performed while preserving the facet joint. (**C**) Postoperative axial T2-weighted MRI demonstrating successful removal of the offending herniated disc and lumbosacral ligament, along with partial resection of the pseudoarticulation, resulting in significant expansion of the L5 nerve root space.

The procedure was technically successful with minimal tissue trauma. Postoperative three-dimensional Computed Tomography (CT) scans confirmed precise bony decompression without compromising the integrity of the facet joint (Figure 4A,B). Postoperative MRI demonstrated successful removal of the HIVD, LSL, and partial pseudoarticulation, with significant expansion of the L5 nerve root space (Figure 4C). The patient’s recovery was rapid: ambulation was achieved on postoperative day 1, and he was successfully discharge on postoperative day 2.

At the 6-month follow-up, the patient reported significant symptomatic relief. The Visual Analogue Scale for leg pain decreased from a preoperative score of 8 to 2. Furthermore, the Oswestry Disability Index improved from 60% to 10%, indicating substantial restoration of functional capacity and quality of life.

## 4. Discussion

The management of FOS is technically demanding due to the highly restricted surgical field bounded by bony structures and the vulnerable L5 DRG [11]. UBE has been increasingly adopted to address the technical challenges of conventional procedures and may offer improved visualization and greater operating flexibility compared to microscopic or uniportal approaches [12].

Although hypertrophied LSL is not the underlying cause in all cases of Far-Out Syndrome, its removal is often an important step in achieving adequate decompression of the L5 nerve root. While en bloc resection techniques have been previously described for ligamentum flavum removal in central canal decompression, their application to the lumbosacral ligament in the extraforaminal region has not been specifically reported. Therefore, the novelty of this technique lies not in the concept of en bloc resection itself, but in its application to the lumbosacral ligament and its integration into the surgical workflow of extraforaminal decompression.

The en bloc LSL resection technique is hypothesized to improve surgical safety by reducing direct manipulation of neural structures. Its rationale is based on a modification of surgical sequencing. Conventional piecemeal techniques typically treat the ligament as the primary barrier requiring fragmentation to access underlying neural elements. In contrast, the en bloc technique prioritizes bony release, allowing indirect mobilization of the ligament before resection. This approach may alter the biomechanical tension of the LSL, facilitating its removal.

This sequence conceptually parallels strategies described in central canal decompression. Previous studies on UBE unilateral laminotomy for bilateral decompression have suggested that en bloc removal of the ligamentum flavum may reduce the incidence of dural tears and nerve root injury compared to piecemeal techniques. In the event of an incidental dural tear, management in endoscopic spine surgery differs from that in open procedures. Soma et al. reported that most cases can be managed conservatively without primary suturing, with only 2.5% requiring direct repair and a very low incidence of pseudomeningocele formation [13].

The ligamentum flavum, like the LSL, may act as a protective barrier. Maintaining its structural integrity until bony attachments are released may reduce premature neural exposure and limit direct contact with surgical instruments. Accordingly, applying the en bloc “lift-and-remove” approach to the LSL may help recreate a protective working environment in the extraforaminal zone and potentially reduce DRG manipulation [9]. These technical advantages may potentially translate into reduced postoperative dysesthesia, although this hypothesis requires validation in future clinical studies.

The en bloc removal of the LSL may offer technical advantages by prioritizing bony preparation before ligament resection. Initial osteoclastic release expands the working corridor and allows improved visualization of the ligament. Once this corridor is established, en bloc removal may provide a wider operative field, which may enhance maneuverability during decompression.

This approach may also reduce repeated instrument insertion and withdrawal and help maintain a more stable irrigation environment. A more stable operative field may improve procedural efficiency and could potentially reduce the risk of retroperitoneal fluid accumulation associated with pressure fluctuations. Although the osteoclastic release phase is technically demanding, subsequent en bloc removal may consolidate multiple steps into a more controlled maneuver.

In addition, the risk of postoperative instability appears to be low. A recent systematic review by Park et al. reported an incidence of less than 1% following biportal endoscopic spine surgery [14].

This study has several limitations. First, it represents a technical report and is not a comparative study. No formal clinical outcome series is presented, and postoperative dysesthesia was not systematically evaluated. In addition, quantitative data regarding complication rates are not provided. Therefore, the proposed neuroprotective benefit of this technique remains hypothetical and has not been clinically validated.

Furthermore, the successful execution of this technique requires a steep learning curve, particularly in mastering the use of the high-speed burr near the nerve root. Surgeons should also be aware of potential complications, such as retroperitoneal fluid collection, which may occur if the anterior fascial barrier is violated. To minimize this risk, osteoclastic release should be limited to the posterior bony attachments, with the L5 vertebral body and psoas muscle serving as the ventral boundary.

## 5. Conclusions

Unilateral biportal endoscopy combined with en bloc resection of the lumbosacral ligament represents a technical modification in the surgical management of Far-Out Syndrome. By prioritizing osteoclastic release of the LSL’s bony attachments prior to ligament removal, this approach may help expand the far-lateral working corridor and reduce direct mechanical manipulation of the L5 dorsal root ganglion. This technique is hypothesized to offer a potential neuroprotective advantage and may contribute to reduced postoperative dysesthesia by minimizing DRG manipulation. However, these potential clinical benefits require validation in future comparative studies.

## Data Availability

No new datasets were generated or analyzed during the current study. Data sharing is not applicable to this article.

## Supplementary Materials

The following supporting information can be downloaded at: https://www.mdpi.com/article/10.3390/jcm15103765/s1, Video S1: Unilateral biportal endoscopic en bloc resection of the lumbosacral ligament following bony release. The video demonstrates the stepwise osteoclastic release of the lumbosacral ligament, establishment of the extraforaminal working corridor, and en bloc removal of the ligament under direct endoscopic visualization.

## Abbreviations

The following abbreviations are used in this manuscript:

UBE	Unilateral Biportal Endoscopy
LSL	Lumbosacral Ligament
DRG	Dorsal Root Ganglion
FOS	Far-Out Syndrome
TP	Transverse Process
SAP	Superior Articular Process
RF	Radiofrequency

## References

[1] Wiltse L.L., Guyer R.D., Spencer C.W., Glenn W.V., Porter I.S. (1984). Alar transverse process impingement of the L5 spinal nerve: The far-out syndrome. Spine.

[2] Chen K.T., Chen C.M. (2025). Anatomy and Pathology of the L5 Exiting Nerve in the Lumbosacral Spine. J. Minim. Invasive Spine Surg. Tech..

[3] Park M.K., Son S.K., Park W.W., Choi S.H., Jung D.Y., Kim D.H. (2021). Unilateral biportal endoscopy for decompression of extraforaminal stenosis at the lumbosacral junction: Surgical techniques and clinical outcomes. Neurospine.

[4] Kavishwar R.A., Liang Y., Lee D., Kim J., Pedraza M., Kim J.S. (2024). O-Arm navigation-guided unilateral biportal endoscopic decompression of far-out syndrome. Neurospine.

[5] Nathan H., Weizenbluth M., Halperin N. (1982). The lumbosacral ligament (LSL), with special emphasis on the “lumbosacral tunnel” and the entrapment of the 5th lumbar nerve. Int. Orthop..

[6] Yang Y.C., Hsieh M.H., Chien J.T., Liu K.C., Yang C.C. (2024). Outcomes of FETD versus UBE in the treatment of L5S1 foraminal stenosis: A comparative study. Heliyon.

[7] Lewandrowski K.U., Dowling Á., Calderaro A.L., dos Santos T.S., Bergamaschi J.P.M., León J.F.R., Yeung A. (2020). Dysesthesia due to irritation of the dorsal root ganglion following lumbar transforaminal endoscopy: Analysis of frequency and contributing factors. Clin. Neurol. Neurosurg..

[8] Epstein N.E. (2016). More nerve root injuries occur with minimally invasive lumbar surgery, especially extreme lateral interbody fusion: A review. Surg. Neurol. Int..

[9] Tumialán L.M. (2023). En bloc resection of ligamentum flavum with laminotomy of the caudal lamina in the minimally invasive laminectomy: Surgical anatomy and technique. Neurosurg. Focus.

[10] Park J., Ahn D.K., Choi D.J. (2024). Treatment concept and technical considerations of biportal endoscopic spine surgery for lumbar spinal stenosis. Asian Spine J..

[11] Kim H.S., Kim J.Y., Wu P.H., Jang I.T. (2021). Effect of dorsal root ganglion retraction in endoscopic lumbar decompressive surgery for foraminal pathology: A retrospective cohort study of interlaminar contralateral endoscopic lumbar foraminotomy and discectomy versus transforaminal endoscopic lumbar foraminotomy and discectomy. World Neurosurg..

[12] Yu A., Li S.Q., Ndjonko L., Frost J.B., Berman D., Park H.-J., Cho S.K. (2025). Comprehensive review of biportal endoscopic spine surgery: History, techniques, and implications in minimally invasive spine surgery. Spine.

[13] Soma K., Kato S., Oka H., Matsudaira K., Fukushima M., Oshina M., Koga H., Takano Y., Iwai H., Ganau M. (2019). Influence of incidental dural tears and their primary microendoscopic repairs on surgical outcomes in patients undergoing microendoscopic lumbar surgery. Spine J..

[14] Park D.Y., Upfill-Brown A., Curtin N., Hamad C.D., Shah A., Kwon B., Heo D.H., Park C.W., Sheppard W.L. (2023). Clinical outcomes and complications after biportal endoscopic spine surgery: A comprehensive systematic review and meta-analysis of 3673 cases. Eur. Spine J..

